# Trends in use of alcohol-free or low alcohol drinks in attempts to reduce alcohol consumption in Great Britain, 2020-2024: a population-based study

**DOI:** 10.1136/bmjph-2025-002775

**Published:** 2025-09-23

**Authors:** Vera Buss, Dimitra Kale, Melissa Oldham, Lion Shahab, Abigail Stevely, Inge Kersbergen, Jamie Brown

**Affiliations:** 1 Behavioural Science and Health, UCL, London, UK; 2 SPECTRUM Consortium, Edinburgh, UK; 3 School of Medicine and Population Health, The University of Sheffield, Sheffield, UK

**Keywords:** trends, Sociodemographic Factors, Cross-Sectional Studies, Public Health, Prevalence

## Abstract

**Introduction:**

Sales and availability of alcohol-free and low alcohol drinks have increased in the UK since 2020. This study aimed to assess trends in the use of alcohol-free and low alcohol drinks to reduce alcohol consumption among people who drink at increasing and higher risk in Great Britain. The study compared trajectories across different subgroups from 2020 to 2024.

**Methods:**

Data were drawn from the Smoking and Alcohol Toolkit Study, which surveys adults monthly across Great Britain about their drinking behaviour. The study included 9397 adults with an AUDIT-C score of 5 or above who attempted to reduce their alcohol consumption in the past year. The analysis used regression analyses to assess time trends in using alcohol-free and low alcohol drinks to cut down overall and among subgroups (eg, gender and age), and in using evidence-based support compared with alcohol-free and low alcohol drinks in attempts to cut down alcohol consumption.

**Results:**

The proportion reporting the use of alcohol-free and low alcohol drinks to reduce alcohol consumption increased from 35.0% (95% confidence interval (CI): 31.8, 38.4) in October 2020 to 43.9% (95% CI: 40.9, 46.9) in August 2024 in serious attempts and from 25.5% (95% CI: 23.2, 28.0) to 38.8% (95% CI: 37.2, 40.4) in any attempt to cut down. Among subgroups, trajectories were mostly comparable. Noticeably, older adults first had lower prevalence of using alcohol-free and low alcohol drinks than young and middle-aged adults but had larger increases over time. While the proportion of participants using alcohol-free and low alcohol drinks in attempts to cut down consumption increased, the proportion using neither alcohol-free/low alcohol drinks nor evidence-based support decreased, and the proportion using evidence-based support, either alone or in combination with alcohol-free/low alcohol drinks, remained low.

**Conclusion:**

The growing use of alcohol-free/low alcohol drinks to reduce alcohol consumption among people at risk of increasing and higher risk drinking in Great Britain highlights the urgent need for more research to establish their effectiveness for alcohol reduction and to inform public health policy. While the use of alcohol-free/low alcohol drinks to cut down rose, the use of evidence-based support remained limited.

**WHAT IS ALREADY KNOWN ON THIS TOPIC**

- People report using alcohol-free and low alcohol drinks as a strategy to reduce their alcohol consumption.

**WHAT THIS STUDY ADDS**

- Between 2020 and 2024, there was a notable increase in the use of alcohol-free and low alcohol drinks in attempts to reduce alcohol consumption among adults in Great Britain who consume alcohol at risky levels. The increase was particularly pronounced among older people, equalling out age differences.

**HOW THIS STUDY MIGHT AFFECT RESEARCH, PRACTICE OR POLICY**

- As the use of alcohol-free and low alcohol drinks continues to rise, it is crucial to establish a robust evidence base for these beverages to support reduction efforts and guide public health messaging.

## Introduction

 Sales of alcohol-free and low alcohol drinks – defined as alcoholic drinks containing up to 1.2% alcohol by volume – in the UK have increased since 2020.^1 2^ Simultaneously, the availability of alcohol-free and low alcohol drinks has increased with more products entering the UK market and more on-trade outlets offering alcohol-free and low alcohol drink options.^1^ The UK Government promotes the substitution of standard alcoholic drinks with alcohol-free/low alcohol drinks as a harm reduction tool.^3^ However, it is not clear to what extent the increasing supply of alcohol-free/low alcohol drinks has contributed to reduction attempts. This study aims to assess trends in the use of alcohol-free/low alcohol drinks to reduce alcohol consumption in Great Britain between 2020 and 2024.

In many social settings in the UK, alcohol consumption is deeply embedded in cultural norms and poses a challenge for people who want to reduce their consumption or abstain altogether.^4^ The pressure to conform, for example, at birthday parties, weddings, or after-work events, can lead to discomfort or exclusion for those choosing not to drink.^5 6^ Alcohol-free/low alcohol drinks may provide a socially acceptable alternative, allowing people to participate in such occasions without feeling negatively judged or having to justify not drinking.^6 7^

Nevertheless, the potential impact of alcohol-free/low alcohol drinks on alcohol consumption and public health remains a subject of debate. Two potential mechanisms have been proposed: on the one hand, consumption may decline if alcohol-free/low alcohol drinks substitute standard-strength alcoholic beverages; on the other hand, overall consumption may remain unchanged if alcohol-free/low alcohol drinks are consumed in addition to regular drinks.^8^ There is also concern that alcohol-free/low alcohol drinks could normalise drinking behaviour in settings where alcohol is typically absent, such as workplaces or gyms, potentially expanding the contexts in which drinking occurs.^9^ Furthermore, some experts have raised concerns about possible initiation or relapse effects, particularly among vulnerable groups such as children and individuals in recovery from alcohol dependence.^10^,^12^ In the UK, guidelines from the National Institute for Health and Care Excellence (NICE) recommend behavioural support or pharmacotherapy for individuals aiming to reduce alcohol consumption.^13^ Pharmacotherapy is prescribed for people with moderate or severe alcohol dependence. Treatment goals vary based on the severity of alcohol use disorder; for risky drinking or mild dependence, reduction of drinking may be recommended, whereas abstinence is advised for moderate or severe dependence or those with comorbidities.^13^ According to a survey in 2014/2015, 13% of people who drink at increasing and higher risk in England attempted to reduce their alcohol consumption in the previous year, with 15% of those using aids such as pharmacotherapy or behavioural support.^14^

An online survey by Alcohol Change UK in 2022 found that 83% of participants drinking at risky levels reported that alcohol-free drinks played an important role in attempts to reduce consumption.^10^ In the survey, people of all ages, genders and income levels found alcohol-free drinks helpful in trying to reduce alcohol consumption.^10^ In the US, an online survey showed that 29% of respondents had used alcohol-free/low alcohol drinks in the past year, with 33% aiming to reduce or abstain from alcohol.^15^ This reason was positively associated with having an alcohol use disorder. However, the frequency and quantity of alcohol-free/low alcohol drinks consumption were also positively associated with the frequency and quantity of alcohol consumption.^15^ In Germany, an online survey revealed that 26% of individuals who were treated for alcohol use disorder, 21% of those with risky alcohol consumption and 19% of those with low-risk alcohol consumption had tried to reduce their alcohol intake with alcohol-free beer.^16^ Among these three groups, 56%, 90% and 95%, respectively, reported successful attempts.^16^

British household purchase data indicated an increase in zero-alcohol beer (alcohol by volume=0.0%) purchases between 2015 and 2020, particularly among younger and more socioeconomically advantaged groups.^17^ In the same period, there was no noteworthy difference for low-alcohol beer (alcohol by volume >0.0% and ≤3.5%) purchases.^17^ A cross-sectional survey study in Great Britain in 2022/23 found that 31% of people aged 16 years and over had consumed alcohol-free/low alcohol drinks, with 10% consuming them weekly.^18^ Consumption was more common among people drinking at risky levels compared with those not drinking, younger compared with older individuals, people from more advantaged compared with less advantaged socioeconomic positions, and people living in South-East England (a more affluent region) compared with Scotland or Yorkshire and the Humber (less affluent regions).^18^

This study aimed to assess trends from 2020 to 2024 in attempts to reduce drinking by consuming alcohol-free/low alcohol drinks among people drinking at risky levels. The study also investigated time trends in the use of alcohol-free/low alcohol drinks in serious attempts to permanently reduce alcohol consumption as trends might differ from those in any attempt. Additionally, the study examined trends across different subgroups (by age, gender, socioeconomic position, nation, alcohol consumption level and additional use of evidence-based support to cut down). Previous research has highlighted differences in the consumption and effectiveness of alcohol-free/low alcohol drinks as harm reduction tools by gender, age, socioeconomic position and region in England.^17^,^19^ Understanding these trends is crucial from a public health perspective because divergent trends could contribute to maintaining health inequalities. The study also compared the profiles of people drinking at risky levels using alcohol-free/low alcohol drinks to reduce consumption over time, assessing whether their sociodemographic and drinking characteristics had changed.

Furthermore, the study compared the profiles of people at risk using: (i) alcohol-free/low alcohol drinks and evidence-based support; (ii) alcohol-free/low alcohol drinks without evidence-based support; (iii) evidence-based support without alcohol-free/low alcohol drinks; or (iv) neither (ie, unaided or using other support such as hypnotherapy or acupuncture). Given there is no conclusive evidence on whether alcohol-free/low alcohol drinks are an effective reduction tool, from a public health perspective, it would be concerning if a rise in the use of alcohol-free/low alcohol drinks to reduce alcohol consumption meant people at risk relied even less on evidence-based methods. Furthermore, it is important to consider the impact of alcohol-free/low alcohol drinks on different populations, particularly people who are dependent on drink. Research suggests that alcohol-free and low alcohol drinks might increase cravings and the desire to drink among people with alcohol use disorder.^20^ Even if alcohol-free and low alcohol drinks are shown to be effective in helping people who are dependent on drink to cut down, combining them with other support, such as behavioural therapy, is advisable, especially for severe alcohol use disorders to help manage negative emotional states and cravings.^21^

Specifically, the study addressed the following research questions (RQs) among adults drinking at increasing and higher-risk levels (operationalised as an AUDIT-C score of 5 or above)^22^ who attempted to restrict their consumption in the past year in Great Britain between 2020 and 2024:

1. Have there been changes over time in the proportion who reported using alcohol-free and low alcohol drinks in any or specifically serious attempts to reduce alcohol consumption?
2. To what extent have changes in any attempts differed by age, gender, socioeconomic position, nation and alcohol consumption level?
3. Has the sociodemographic and drinking profile among those using alcohol-free/low alcohol drinks in attempts to reduce alcohol consumption changed over this period?
4. Have there been changes over time in the proportion who tried to restrict their consumption in the past year by using (i) alcohol-free/low alcohol drinks and evidence-based support; (ii) alcohol-free/low alcohol drinks but not evidence-based support; (iii) evidence-based support but not alcohol-free/low alcohol drinks; or (iv) neither?

## Methods

### Study design and participants

The study used data collected in Great Britain between October 2020 (start of data collection in Scotland and Wales) and August 2024 (most recent data) as part of the Smoking and Alcohol Toolkit Study, which collects monthly data about adults’ sociodemographic, smoking and drinking characteristics.^23^,^25^ Every month, approximately 2450 households are selected from 227 403 output areas, each comprising of roughly 300 households. The sampling strategy consists of a hybrid of random location and quota sampling. Areas are stratified by an established geo-demographic classification of the British population. A market research company conducts phone interviews until the monthly quotas are fulfilled. The research team had access to only anonymised data. Ethics approval for the Smoking and Alcohol Toolkit Study was provided by the University College London Ethics Committee (ID 0498/001). The manuscript follows the Strengthening the Reporting of Observational Studies in Epidemiology (STROBE) statement.^26^ The study protocol was pre-registered and the statistical code and dataset are available from the Open Science Framework repository.^27 28^

We included people aged 18 years and older, which is the legal age of sale of alcohol in the UK. Further, we limited the sample to adults drinking at increasing and higher-risk levels, operationalised as an AUDIT-C score of 5 or above,^22^ and having tried to cut down their alcohol consumption in the previous year, including those who are currently making an attempt (*“Are you currently trying to restrict your alcohol consumption, for example, by drinking less, choosing lower strength alcohol or using smaller glasses?”* and *“How many attempts to restrict your alcohol consumption have you made in the last 12 months (e.g. by drinking less, choosing lower strength alcohol or using smaller glasses)? Please include all attempts you have made in the last 12 months, whether or not they were successful, AND any attempt that you are currently making.”*). These questions about attempting to cut down were not asked every wave in England. Therefore, we excluded waves in which the questions were not asked across all of Great Britain.

### Outcome measures and covariates

All measures were self-reported. Use of alcohol-free/low alcohol drinks in any attempt to cut down was classified based on the following question: *“Which, if any, of the following did you use to try to help restrict your alcohol consumption during the most recent attempt?”*. The variable was coded as ‘yes’ if someone reported using “Low-alcohol/Alcohol-free drinks” to help them cut down, and otherwise as ‘no’. All answer options included (multiple answer options possible): (*1) Any medicines (eg, acamprosate (Campral), disulfiram (Antabuse), nalmefene (Selincro); (2) Attended one or more one-to-one or group counselling/advice/support sessions for help with drinking; (3) Attended a specialist alcohol clinic or centre for help with drinking; (4) Consulted a community pharmacist for help with drinking; (5) Phoned a helpline for help with drinking (eg,DrinkLine); (6) An alcohol self-help book or booklet; (7) Visited a website for help with drinking; (8) Used an alcohol application ('app') on a handheld computer (smartphone, tablet, PDA); (9) Hypnotherapy for help with drinking; (10) Acupuncture for help with drinking; (11) Low-alcohol/Alcohol-free drinks; (12) Other (please specify*).

We classified attempts in which someone reported using alcohol-free and low alcohol drinks to help them cut down as serious if they previously stated that their most recent attempt to restrict their alcohol consumption was a serious attempt to permanently cut down on drinking: *“During your most recent attempt to restrict your alcohol consumption, was it a serious attempt to cut down on your drinking permanently?”*.

Further, we used survey wave as a continuous variable ranging from 1 (October 2020) to 47 (August 2024), modelled using restricted cubic splines to allow for non-linear trends.^29 30^ As sociodemographic characteristics, we included age (continuous variable, modelled using restricted cubic splines), gender (binary variable, women or men; when reporting characteristics of study participants, we also provided the proportion who identify as non-binary, but these were excluded when stratifying by gender due to small numbers), socioeconomic position measured using the National Readership Survey’s classification of social grade^31^ (ordinal variable, AB: higher and intermediate managerial, administrative and professional; C1C2: supervisory, clerical and junior managerial, administrative and professional, skilled manual workers; DE: semi-skilled and unskilled manual workers, state pensioners, casual and lowest grade workers, unemployed with state benefits only), nation (categorical variable split into England, Scotland and Wales). We retrieved estimates for specific ages (18, 25, 35, 45, 55, 65 and 75 years) from the model using spline functions.

To account for differences in drinking behaviour, we included alcohol consumption level based on the AUDIT-C score^22^ (ordinal variable, increasing risk: AUDIT-C 5–7; higher risk: AUDIT-C 8–10; possible dependence: AUDIT-C 11–12). To measure whether someone used evidence-based support to restrict consumption, we used the same question as for the outcome measure (see above). If someone reported using medication or behavioural support to help them cut down their consumption (answer options 1 to 8 for question above), they were classified as using evidence-based support.^1332^,^35^

### Analysis

A complete case analysis was conducted. Responses collected as “Don’t know” or “Refused” were classified as missing. As outlined above, surveys without data collection on attempts to cut down across all of Great Britain were excluded from the study. However, as we modelled time using restricted cubic splines, we were able to extract estimates for each month from the model, including those in which no data collection took place. The analysis was conducted on weighted data using raking^36^ to match the population of Great Britain. Unweighted results are in the online supplemental file 1. The analysis was conducted in RStudio (version 2022.07.2, R version 4.2.1).

For RQ1, we assessed time trends in the prevalence of using alcohol-free/low alcohol drinks to reduce alcohol consumption among people at increasing and higher-risk of drinking who tried to restrict their consumption in the past year using log binomial regression. Additionally, we assessed the prevalence of using alcohol-free/low alcohol drinks in serious attempts to permanently cut down on drinking. The time trends were depicted graphically and further estimates for October 2020 (beginning of the time series) and August 2024 (end of the time series) were compared by calculating prevalence ratios (PRs) and their 95% confidence intervals (CIs), which were calculated using bootstrapping (n=2000). The standard errors of the estimates were retrieved from the bootstrap samples and then used to compute the 95% CIs.

For RQ2, we ran five additional models with alcohol-free/low alcohol drinks in attempts to cut down as the outcome and an interaction term between time and (i) age, (ii) gender, (iii) socioeconomic position, (iv) nation, or (v) alcohol consumption level. The stratified time trends were presented graphically and the modelled estimates for October 2020 and August 2024 compared for each subgroup by calculating PRs and 95% CIs.

For RQ3, we compared the sociodemographic and drinking profiles of those who reported attempting to reduce their alcohol consumption with alcohol-free and low alcohol drinks over time by providing descriptive statistics of their age, gender, socioeconomic position, the nation they live in, and their alcohol consumption in different time periods (October 2020 to September 2021, October 2021 to September 2022, October 2022 to September 2023, October 2023 to August 2024).

For RQ4, we descriptively assessed time trends (from October 2020 to August 2024) in the proportion of people at increasing and higher-risk of drinking who tried to restrict their consumption in the past year by using (i) alcohol-free/low alcohol drinks and evidence-based support, (ii) alcohol-free/low alcohol drinks but not evidence-based support, (iii) evidence-based support but not alcohol-free/low alcohol drinks, or (iv) neither. We retrieved estimates from a multinomial logistic regression model and calculated their 95% CIs drawing n=2000 samples of the covariance matrix following the multivariate normal distribution and then taking the 2.5^th^ and 97.5^th^ percentile of the estimated probabilities.

### Patient and public involvement

There are several meetings a year with a Patient and Public Involvement group to discuss the Alcohol Toolkit Study from which data were drawn for the present study. The interviewers who collect the survey data also collect feedback from participants on the questions included in the Alcohol Toolkit Study survey, particularly regarding clarifications of questionnaire items. The research idea for this study was shaped by discussions with public health advocacy groups and the public. Patients and the public were not directly involved in the design or conduct of this study.

## Results

In total, the dataset included 9429 (unweighted) participants who drank at increasing and higher-risk and attempted to cut down their consumption in the previous year. Of these, 32 had missing values for gender (n=15, 0.2%) or use of alcohol-free/low alcohol drinks in attempts to cut down (n=17, 0.2%). After these participants were excluded, the sample comprised 9397 (unweighted) participants (see online supplemental figure S1 to show how sample size was derived). A summary of participants’ characteristics is provided in table 1. To provide more context, online supplemental table S1 shows characteristics of the whole population surveyed at the time of this study and stratified by AUDIT-C score ≥5.

**Table 1:** Characteristics of included adults who drink at increasing and higher risk who made a past-year cut-down attempt (N_unweighted_=9397)

Characteristic	Weighted estimate
Age, median in years (IQR)	46 (33, 58)
Women, % (95% CI)	40.4 (39.3, 41.5)
Men, % (95% CI)	58.8 (57.7, 59.9)
Non-binary, % (95% CI)	0.7 (0.6, 0.9)
Social grades AB, % (95% CI)	33.9 (33.0, 34.9)
Social grades C1C2, % (95% CI)	50.3 (49.2, 51.5)
Social grades DE, % (95% CI)	15.8 (15.0, 16.6)
England, % (95% CI)	86.1 (84.6, 87.6)
Scotland, % (95% CI)	9.0 (8.0, 10.0)
Wales, % (95% CI)	4.9 (4.3, 5.5)
AUDIT-C 5–7, % (95% CI)	58.6 (57.2, 60.0)
AUDIT-C 8–10, % (95% CI)	35.2 (33.9, 36.5)
AUDIT-C 11–12, % (95% CI)	6.2 (5.6, 6.7)
Serious cut-down attempt, % (95% CI)	33.3 (32.2, 34.4)

Social grades AB indicate most advantaged and DE least advantaged socioeconomic positions.

Alcohol consumption levels categorised as increasing risk: AUDIT-C 5-7; higher risk: AUDIT-C 8-10; possible dependence: AUDIT-C 11-12.

IQR, interquartile range.

### RQ1: Using alcohol-free/low alcohol drinks in any or serious attempts

**Figure 1:**
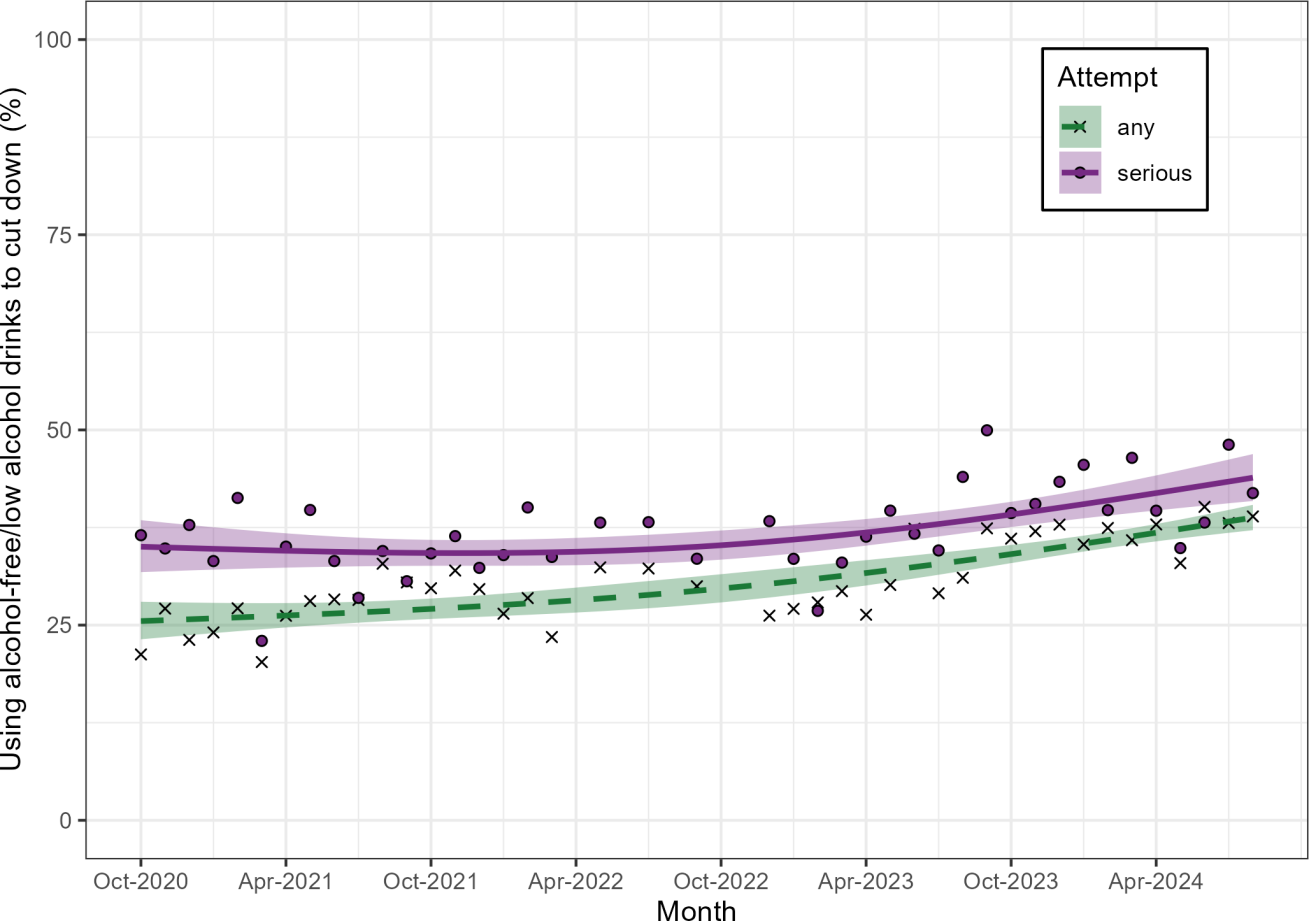
Time trends (2020–2024) in the prevalence of using alcohol-free/low alcohol drinks in any or serious attempts to reduce alcohol consumption among people at increasing and higher-risk of drinking who tried to restrict their consumption in the past year, modelled using restricted cubic splines. Shaded areas represent 95% CIs. Dots and crosses represent unmodelled estimates.

At the start of the time series in October 2020, the proportion of people reporting the use of alcohol-free/low alcohol drinks in serious cut-down attempts (35.0%, 95% CI: 31.8, 38.4) was higher than in any attempt (25.5%, 95% CI: 23.2, 28.0; figure 1 and table 2). However, over time, the difference became smaller (August 2024, serious: 43.9%, 95% CI: 40.9, 46.9 vs any: 38.8%, 95% CI: 37.2, 40.4) due to a more pronounced increase in any (PR: 1.52, 95% CI: 1.36, 1.69) compared with serious attempts (PR: 1.25, 95% CI: 1.12, 1.40).

**Table 2:** Modelled estimates for October 2020 and August 2024 and corresponding prevalence ratios for using alcohol-free and low alcohol drinks to cut down consumption among people at increasing and higher-risk of drinking in serious or any attempt and in any attempt stratified by sociodemographic and drinking characteristics

	Oct 20, % (95% CI)	Aug 24, % (95% CI)	PR (95% CI)
In serious attempt	35.0 (31.8, 38.4)	43.9 (40.9, 46.9)	1.25 (1.12, 1.40)
In any attempt	25.5 (23.2, 28.0)	38.8 (37.2, 40.4)	1.52 (1.36, 1.69)
Age 18	26.5 (18.9, 35.9)	31.2 (23.4, 40.2)	1.18 (0.81, 1.70)
Age 25	27.3 (21.8, 33.7)	34.2 (29.3, 39.4)	1.25 (0.93, 1.68)
Age 35	27.9 (24.5, 31.6)	38.2 (35.9, 40.4)	1.37 (1.06, 1.76)
Age 45	27.4 (24.3, 30.6)	41.0 (37.0, 45.0)	1.50 (1.16, 1.94)
Age 55	25.1 (22.8, 27.4)	41.9 (38.0, 46.0)	1.67 (1.31, 2.14)
Age 65	21.5 (19.0, 24.2)	41.1 (37.6, 44.6)	1.91 (1.54, 2.38)
Age 75	17.4 (12.7, 23.3)	38.9 (31.8, 46.6)	2.24 (1.76, 2.85)
Women	26.6 (23.7, 29.7)	41.8 (37.6, 46.1)	1.57 (1.35, 1.83)
Men	24.7 (22.1, 27.5)	36.9 (33.2, 40.8)	1.49 (1.28, 1.74)
Social grades AB	27.9 (24.9, 31.2)	46.4 (43.3, 49.6)	1.66 (1.42, 1.95)
Social grades C1C2	25.1 (21.7, 29.0)	36.9 (33.2, 40.8)	1.47 (1.22, 1.77)
Social grades DE	21.0 (14.7, 29.1)	27.5 (19.9, 36.7)	1.31 (0.92, 1.86)
England	25.4 (22.8, 28.1)	39.0 (37.1, 40.9)	1.54 (1.36, 1.73)
Scotland	26.7 (21.7, 32.3)	36.6 (30.9, 42.7)	1.37 (1.14, 1.65)
Wales	26.4 (19.5, 34.7)	40.3 (33.8, 47.2)	1.53 (1.25, 1.86)
AUDIT-C 5–7	25.1 (22.6, 27.8)	41.7 (39.0, 44.5)	1.66 (1.46, 1.89)
AUDIT-C 8–10	26.1 (23.1, 29.4)	32.6 (27.8, 37.8)	1.25 (1.02, 1.52)
AUDIT-C 11–12	24.4 (13.8, 39.4)	42.5 (30.5, 55.5)	1.74 (1.20, 2.53)

Social grades AB indicate most advantaged and DE least advantaged socioeconomic positions.

Alcohol consumption levels categorised as increasing risk: AUDIT-C 5-7; higher risk: AUDIT-C 8-10; possible dependence: AUDIT-C 11-12.

### RQ2: Using alcohol-free/low alcohol drinks in any attempts across subgroups

**Figure 2:**
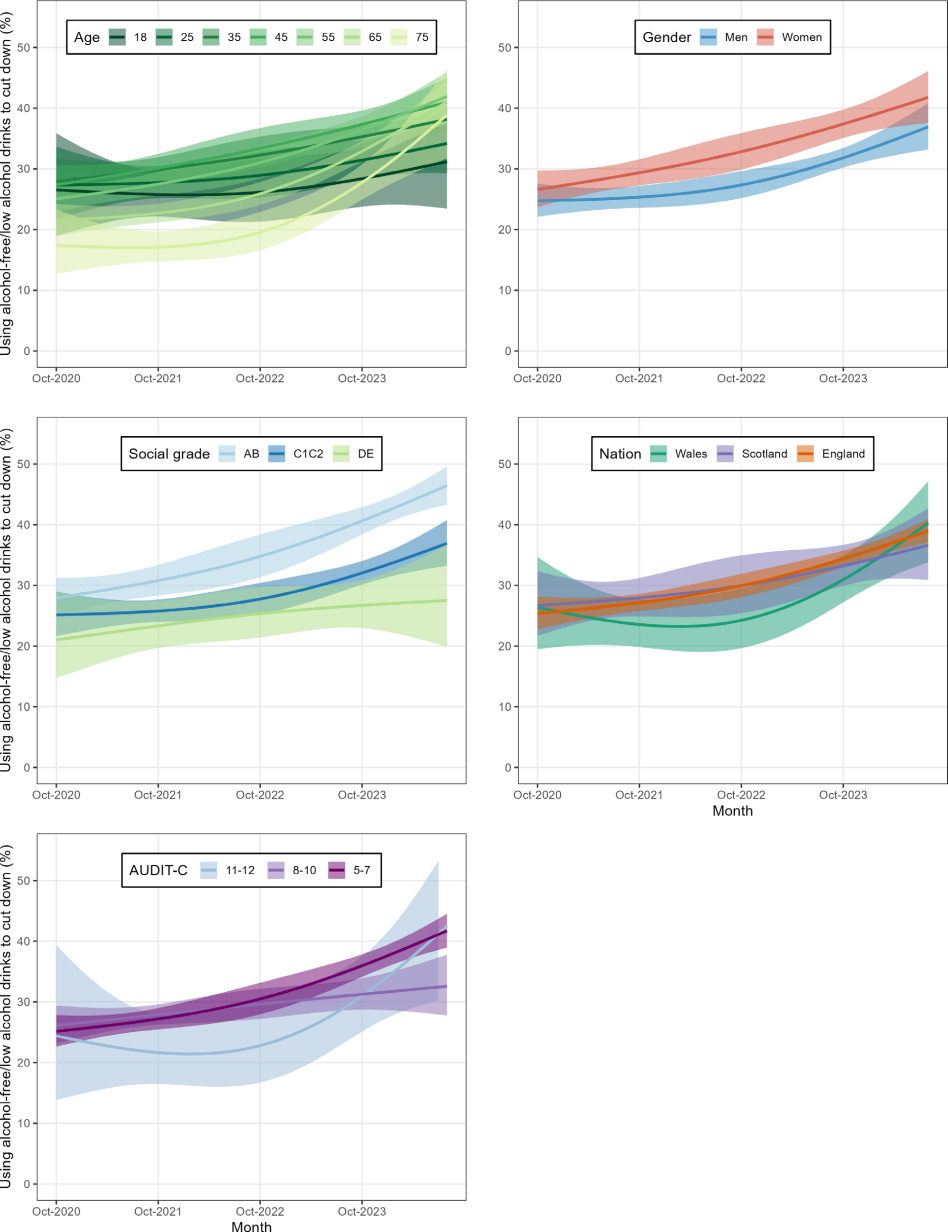
Time trends (2020–2024) in the prevalence of using alcohol-free/low alcohol drinks to reduce alcohol consumption among people at increasing and higher-risk of drinking who tried to restrict their consumption in the past year, stratified by age, gender, social grade, nation, and alcohol consumption level, modelled using restricted cubic splines. Social grades AB indicate most advantaged and DE least advantaged socioeconomic positions. Alcohol consumption levels categorised as increasing risk: AUDIT-C 5–7; higher risk: AUDIT-C 8–10; possible dependence: AUDIT-C 11–12. Shaded areas represent 95% CIs.

At the start of the time series in October 2020, older adults (65+) tended to have a lower prevalence of using alcohol-free/low alcohol drinks in cut-down attempts than young and middle-aged adults, but over time, there was a greater increase in prevalence among older age groups (table 2 and figure 2). Throughout the entire period, women tended to have a higher prevalence of using alcohol-free/low alcohol drinks in cut-down attempts than men, with both groups showing similar increases over time. People with the most advantaged socioeconomic positions (social grades AB) had overall the highest prevalence, and people with the least advantaged socioeconomic positions (social grades DE) had the lowest. The most advantaged socioeconomic positions (social grades AB) may also have experienced a greater increase over time than the other groups. There were no clear differences between nations, although people in Wales may have increased their use of alcohol-free/low alcohol drinks in quit attempts slightly later, around October 2022, than people in England and Scotland. There were also no clear differences between the three alcohol consumption levels, but those drinking at levels of possible dependence might have increased their use of alcohol-free/low alcohol drinks later than those with lower alcohol consumption levels.

### RQ3: Profile of those using alcohol-free/low alcohol drinks to cut down

The sociodemographic and drinking profile of people at increasing and higher-risk of drinking who used alcohol-free/low alcohol drinks to reduce alcohol consumption did not change substantially over time (online supplemental table S2) and was broadly comparable to the overall profile of people with increasing and higher-risk consumption (table 1).

### RQ4: Using alcohol-free/low alcohol drinks or evidence-based support to cut down

**Figure 3:**
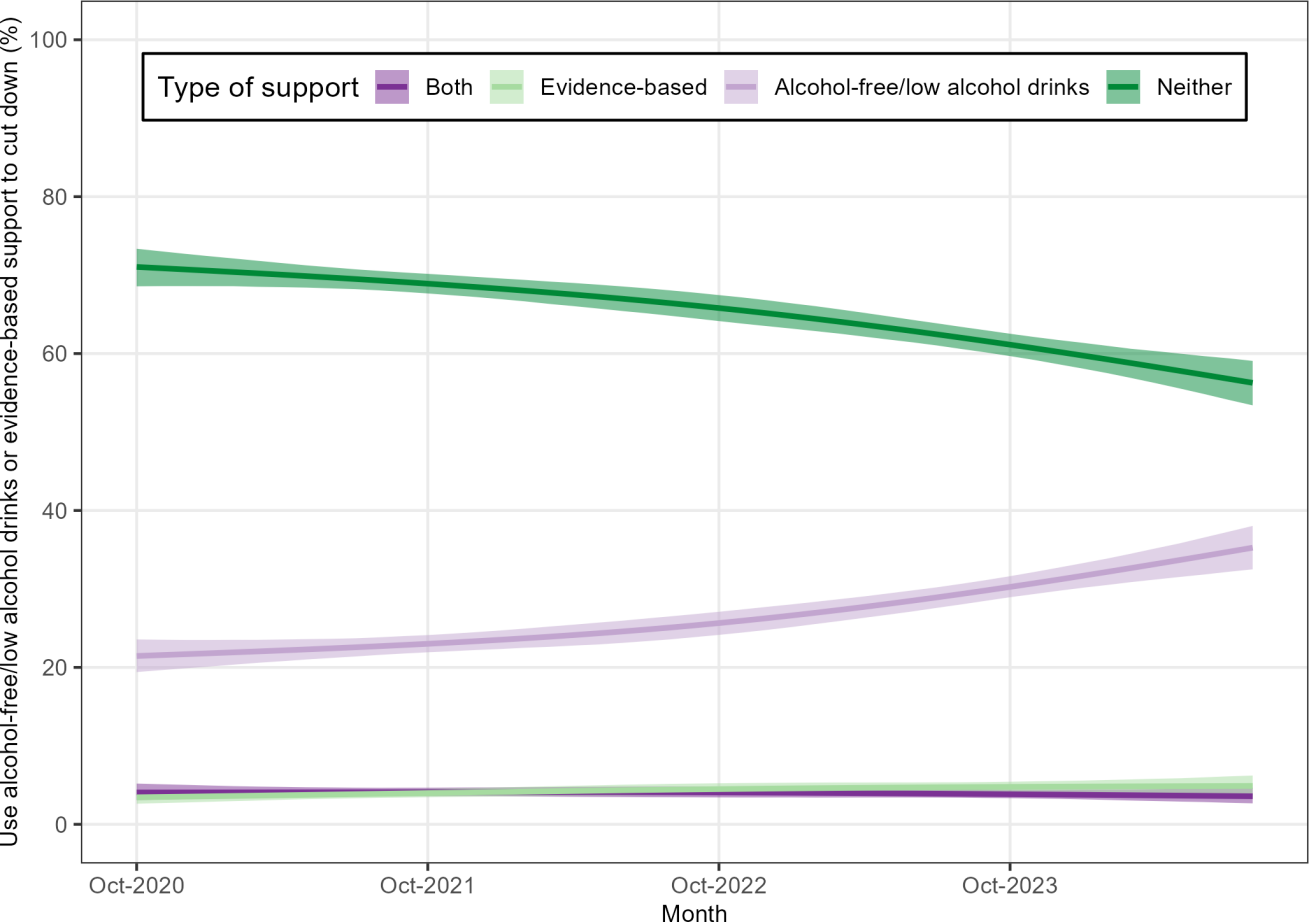
Time trends (2020–2024) in the proportion of people at increasing and higher-risk of drinking who tried to restrict their consumption in the past year by using (**i**) alcohol-free/low alcohol drinks and evidence-based support; (ii) alcohol-free/low alcohol drinks but not evidence-based support; (iii) evidence-based support but not alcohol-free/low alcohol drinks; or (iv) neither, modelled using restricted cubic splines. Shaded areas represent 95% CIs.

Over time, the proportion of people at increasing and higher-risk of drinking who tried to restrict their consumption by using alcohol-free/low alcohol drinks increased while the proportion who used neither alcohol-free/low alcohol drinks nor evidence-based support decreased. However, there was no apparent difference in the proportions using both or evidence-based support (figure 3). Overall, only a small proportion used evidence-based support in cut-down attempts.

### Sensitivity analysis

The results of the unweighted analysis (online supplemental tables S3–S5 and figures S2–S4) were consistent with those of the weighted analysis.

## Discussion

### Summary of results

The use of alcohol-free/low alcohol drinks to reduce alcohol consumption among people at increasing and higher-risk of drinking increased in Great Britain between 2020 and 2024, in both serious and any attempts to cut down. Specifically, the prevalence of using alcohol-free/low alcohol drinks in serious attempts rose from 35% in October 2020 to 44% in August 2024, while the prevalence in any attempt increased from 26% to 39%. The increase was particularly pronounced among older adults (65+) who had a lower prevalence than young and middle-aged adults at the start of the time series but had a greater relative increase over time. Women and people with the most advantaged socioeconomic positions had overall higher prevalence of using alcohol-free/low alcohol drinks to cut down than men and people with less advantaged socioeconomic positions. While the proportion of people trying to cut down their alcohol consumption by using alcohol-free/low alcohol drinks increased, there was a decrease among those using neither alcohol-free/low alcohol drinks nor evidence-based support, but the proportion using evidence-based support either alone or in combination with alcohol-free/low alcohol drinks was overall small and stable (roughly 10%).

### Strengths and limitations

The strengths of this study are the large, population-based sample with few missing values and monthly continuous data collection, allowing for the assessment of trends over a 4-year period. Among the limitations are that data were self-reported. It is also important to consider that AUDIT-C scores were assessed based on alcohol consumption at the time of the interview. Therefore, people who successfully cut down their consumption to a low-risk level (ie, their AUDIT-C score was below 5 at the time of the interview) were not included in the study. Given that this study was cross-sectional, it was not possible to evaluate previous behavioural changes; rather, the focus was on presenting a snapshot of the situation at the time participants were surveyed.

Additionally, the exclusion of certain waves without data collection on relevant variables across all of Great Britain may have biased the results. However, we included survey wave as a variable, modelled using restricted cubic splines, in the regression models which should have mitigated any effects related to missing waves, and the sampling strategy is designed to ensure a representative sample each month. Further, the sample sizes of some subgroups were relatively small, resulting in wide CIs around the estimates.

A further study limitation is that the survey assessed the use of alcohol-free and low alcohol drinks together. Therefore, we are unable to distinguish between the two. One could argue that people using low alcohol drinks to cut down alcohol consumption may differ from those using only alcohol-free drinks to abstain from alcohol completely. Also, when comparing research findings across countries, there are some terminological disparities on what is considered to fall in the alcohol-free as opposed to the low-alcohol category.^37^ Similarly, it is possible that people may consider drinks such as kombucha or essence flavoured sodas as alcohol-free drinks. Therefore, qualitative research might be helpful in further exploring what people understand under the term ‘Low-alcohol/Alcohol-free drinks’.

Further, this study only assessed trends in self-reported use of alcohol-free/low alcohol drinks in alcohol reduction attempts and, therefore, cannot make any assumptions about the effectiveness of alcohol-free/low alcohol drinks in reducing alcohol consumption. As we only included adults in the study, the results are not generalisable to individuals who are underage. However, presumably the proportion of underaged who are drinking at increasing and higher-risk levels and have tried to reduce their consumption in the past year is relatively small.

### Implications

The rise in the prevalence of alcohol-free/low alcohol use in cut-down attempts in Great Britain may be driven by greater availability of alcohol-free/low alcohol drinks in pubs or increased marketing, such as the partnership between the charity organising the Dry January campaign and alcohol-free drink producers which started in 2022.^1 38^ It is also possible that the observed changes are consumer-driven, ie, that people’s interest in alternatives to standard-strength alcoholic drinks is growing, and the market is responding to this demand. In any case, it is vital for future research to establish whether alcohol-free/low alcohol drinks are an effective tool to reduce alcohol consumption.

Currently, it remains unclear whether consuming alcohol-free/low alcohol drinks will actually result in a reduction of alcohol consumption or whether people consume alcohol-free/low alcohol drinks in addition to standard alcoholic drinks, therefore, resulting in no change of alcohol consumption.^8^ Some studies based on household purchasing data suggest that alcohol-free/low alcohol drinks could replace standard-strength alcoholic drinks rather than being consumed in addition to them.^17 39 40^ A recent study combining on-trade and off-trade sales data suggests that alcohol-free/low alcohol drinks consumption remains so limited that it is unlikely to have a substantial public health impact in the UK in 2025.^2^ A randomised controlled trial from Japan, sponsored by an alcohol manufacturer, investigated the potential of non-alcoholic beverages to reduce alcohol consumption among individuals drinking at risky levels.^41^ The intervention group received free non-alcoholic beverages for 12 weeks which resulted in a significant reduction in alcohol consumption compared with the control group.^41^ Subgroup analyses indicated that these reductions were only significant between the control and intervention groups among men and those with AUDIT scores below 15.^19 42^ This echoes the findings of a systematic review including five primary studies of participants with a history of alcohol dependence which proposes that alcohol-free/low alcohol drinks may not be suitable for people who are dependent on drink.^20^ More research on the effects of alcohol-free/low alcohol drinks on people who are dependent on drink would be valuable.

The small gender difference in the prevalence of using alcohol-free/low alcohol drinks to reduce alcohol consumption among people at increasing and higher-risk of drinking who tried to restrict their consumption reported in the current study might be related to women generally expressing a greater health consciousness and tendency to seek healthier food alternatives.^43^ One study found that, for women, health consciousness was a predictor of non-alcoholic beer consumption frequency but not for men.^44^ Another study found that a higher proportion of women than men cited health concerns as their reason for trying to reduce alcohol consumption.^45^ Further, research has indicated that women might be particularly receptive to advertising that refers to the caloric content of alcoholic beverages,^46 47^ which might make alcohol-free/low alcohol drinks attractive to women whose motivation for alcohol reduction is to lose weight. Additionally, some women might choose alcohol-free/low alcohol drinks to reduce their alcohol consumption when they are trying to become pregnant.^9 48^ However, these are tentative explanations that require further investigations. It is also worth noting that in absolute numbers more men than women used alcohol-free/low alcohol drinks to reduce alcohol consumption because more men drink at increasing and higher-risk levels (approximately 60% in the current study).

The difference in age observed at the start of the time series resolved over time, but differences by socioeconomic status remained. The age changes could be related to younger age groups being more likely to try new products, in this case alcohol-free/low alcohol drinks. An analysis of early adopters of new supermarket products found that the proportion spent on new products declined with age.^49^ The study authors argued that younger consumers might be more actively searching for new products, as they have less established taste preferences and brand loyalties. Therefore, new products might also be more advertised to younger people.^49^ Over time, the age gradient may have evened out as the products became more widely available and older age groups began to consume the products.

The socioeconomic divide is potentially concerning because alcohol-related harm is disproportionately experienced by people who are less advantaged.^50 51^ If alcohol-free/low alcohol drinks are shown to be effective for harm reduction, it will be vital to develop interventions targeted at people with less advantaged socioeconomic positions to reduce health inequalities. Other studies have found that people expressed dissatisfaction with the prices of alcohol-free/low alcohol drinks compared with standard alcoholic drinks.^52^ The pricing of alcoholic drinks could be regulated by the government to ensure that alcohol-free/low alcohol drinks are sold at a considerably lower price than their higher strength counterparts. For example, minimum unit pricing in Scotland has been shown to be effective in reducing alcohol-related harm, to positively impact health inequalities, and to shift purchases to lower strength products.^53 54^

The fact that people mostly seem to use alcohol-free/low alcohol drinks without additionally using evidence-based support may be suboptimal. Even if effective, alcohol-free/low alcohol drinks may be more effective if used in combination with other support methods, especially for people with severe alcohol use disorder.^21^ It is notable that the use of alcohol-free/low alcohol drinks was greater among those making a serious attempt to permanently cut down. Findings from interviews with women in recovery showed that consuming alcohol-free/low alcohol drinks was helpful for some during the process of reaching sobriety, while others avoided the products at the beginning and only started using them once they reached a stable state.^55^ The study also highlighted the need for clear labelling of alcohol-free/low alcohol drinks as it is important for people to know the exact alcohol content of the various products (eg, low alcohol content vs alcohol-free).^55^ Currently, different terms are used to describe these drinks, such as “no”, “free”, “zero”, “low”, or “light” and there are variations between jurisdictions.^37^ The use of more standardised descriptors and clearly labelling the exact alcohol content would help people make informed choices and avoid situations where low-alcohol drinks are offered to people who do not want or should not drink alcohol. Generally, only about 1 in 10 reported using evidence-based methods, suggesting that these tools should be better promoted so that people who try to restrict their alcohol consumption are aware of them and believe in their effectiveness.

### Conclusion

The use of alcohol-free/low alcohol drinks to reduce alcohol consumption has significantly increased among people at increasing and higher-risk of drinking in Great Britain between 2020 and 2024. It is now important to establish whether alcohol-free/low alcohol drinks are an effective alcohol reduction tool. If substituting standard-strength alcoholic drinks with alcohol-free/low alcohol alternatives proves effective, targeted interventions should be developed to promote their use among socioeconomically less advantaged groups, where prevalence is lower. While the use of alcohol-free/low alcohol drinks to cut down rose, the use of evidence-based support remained limited, highlighting the need to assess whether alcohol-free/low alcohol drinks can effectively help people to cut down their consumption and can therefore be considered evidence-based support. In the interim, public health campaigns should communicate the potential role of alcohol-free/low alcohol drinks in reducing alcohol consumption based on the best available evidence, while also strengthening awareness and accessibility of proven support services to promote a comprehensive and balanced harm reduction approach.

## Supplementary material

10.1136/bmjph-2025-002775 online supplemental file 1

## Data Availability

Data are available in a public, open access repository.
