# Supplementary material for: Trends in use of alcohol-free or low alcohol drinks in attempts to reduce alcohol consumption in Great Britain, 2020-2024: a population-based study
Source: BMJ Public Health. 2025 Sep 23;3(2):e002775. doi: 10.1136/bmjph-2025-002775 (PMC12458652; doi:10.1136/bmjph-2025-002775)
Supplement: online supplemental file 1 [file bmjph-3-2-s001.pdf]

Supplementary material

Article: Trends in use of alcohol-free or low alcohol drinks in attempts to reduce alcohol consumption in Great Britain, 2020-2024

Table of Contents

1. Outcome measures 1
2. Sample size 2
3. Participant characteristics when including whole population 2
4. Profile of those using alcohol-free/low alcohol drinks to cut down 3
5. Unweighted characteristics of participants 4

1. Outcome measures

Alcohol Use Disorder Identification Test – Consumption (AUDIT-C)

The survey introduced these questions with the following:

"These first few questions ask about the alcohol you have drunk in the last 6 months, including about how many standard drinks you have consumed. Please note that 1 standard drink equals 1 unit of alcohol. So, for example, a small glass of wine or a single measure of spirits is 1 standard drink, while a pint of regular beer or lager is equal to 2 standard drinks or 2 units, and a bottle of wine is equal to 9 units. If you are unsure, please ask me to help you work it out.

Please be aware that all your answers will be handled confidentially."

AUDIT-C question 1: *"How often do you have a drink containing alcohol?*

- i. *Never [value: 0]*
- ii. *Monthly or less [value: 1]*
- iii. *2 to 4 times a month [value: 2]*
- iv. *2 to 3 times a week [value: 3]*
- v. *4 to 5 times a week [value: 4]*
- vi. *6 or more times a week [value: 4]*
- vii. *Don't know*
- viii. *Refused"*

The following questions are asked to all except those who answered (i), (vii), or (viii) to AUDIT-C question 1.

AUDIT-C question 2: *"How many standard drinks containing alcohol do you have on a typical day when you are drinking?*

- i. *1 to 2 [value: 0]*
- ii. *3 to 4 [value: 1]*
- iii. *5 to 6 [value: 2]*
- iv. *7 to 9 [value: 3]*
- v. *10 to 12 [value: 4]*
- vi. *13 to 15 [value: 4]*
- vii. *16 or more [value: 4]*
- viii. *Don't know*
- ix. *Refused"*

AUDIT-C question 3: *"How often do you have six or more standard drinks on one occasion?*

- i. *Never [value: 0]*
- ii. *Less than monthly [value: 1]*
- iii. *Monthly [value: 2]*
- iv. *Weekly [value: 3]*
- v. *Daily or almost daily [value: 4]*
- vi. *Don't know*
- vii. *Refused"*

Increasing and higher-risk drinking – binary variable, measured using the AUDIT-C (i.e., AUDIT questions 1-3), with a score of 5 or above indicating increasing and higher-risk drinking.

2. Sample size

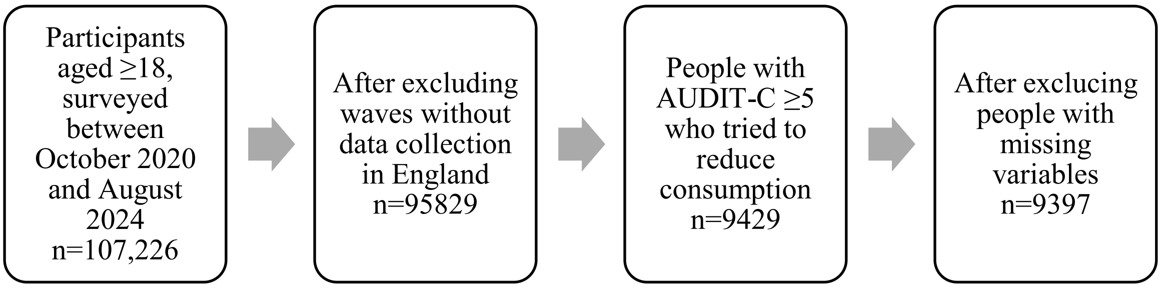

Figure S1: Flowchart showing how sample size was derived.

3. Participant characteristics when including whole population

Table S1: Characteristics of whole population with complete data surveyed at the time of this study ($N_{unweighted}$=92,570) and stratified by AUDIT-C score. Data in the table are weighted.

**Characteristic**	**All adults**	**AUDIT-C <5**	**AUDIT-C ≥5**
Age, median in years (IQR)	49 (33, 63)	50 (33, 65)	46 (32, 59)
Women, % (95% CI)	50.8 (50.7, 50.9)	57.0 (56.7, 57.3)	37.7 (37.2, 38.2)
Men, % (95% CI)	48.6 (48.5, 48.7)	42.4 (42.1, 42.7)	61.5 (61.0, 62.1)
Non-binary, % (95% CI)	0.7 (0.6, 0.7)	0.6 (0.5, 0.7)	0.8 (0.7, 0.9)
Social grades AB, % (95% CI)	26.7 (26.6, 26.8)	24.7 (24.5, 25.0)	30.8 (30.4, 31.3)
Social grades C1C2, % (95% CI)	49.8 (49.7, 49.9)	48.8 (48.6, 49.0)	51.9 (51.3, 52.4)
Social grades DE, % (95% CI)	23.5 (23.4, 23.6)	26.5 (26.2, 26.7)	17.3 (16.8, 17.8)
England, % (95% CI)	86.4 (86.3, 86.4)	86.7 (86.6, 86.9)	85.7 (85.3, 86.0)
Scotland, % (95% CI)	8.7 (8.7, 8.7)	8.3 (8.1, 8.4)	9.6 (9.4, 9.9)
Wales, % (95% CI)	4.9 (4.9, 4.9)	5.0 (4.9, 5.1)	4.7 (4.6, 4.9)
AUDIT-C ≥5, % (95% CI)	32.1 (31.6, 32.6)	–	–

Abbreviations: CI, confidence interval; IQR, interquartile range. Social grades AB indicate most advantaged and DE least advantaged socioeconomic positions. AUDIT-C ≥5 indicates increasing and higher-risk drinking.

4. Profile of those using alcohol-free/low alcohol drinks to cut down

Table S2: Sociodemographic and drinking profile of people drinking at increasing and higher-risk levels who used alcohol-free/low alcohol drinks to reduce alcohol consumption across the years.

	Oct 20 – Sep 21	Oct 21 – Sep 22	Oct 22 – Sep 23	Oct 23 – Aug 24
Age, median (IQR)	44 (33, 55)	44 (34, 54)	47 (34, 59)	46 (33, 58)
Women, % (95% CI)	45.2 (41.9, 48.4)	41.4 (34.8, 47.9)	45.8 (43.5, 48.0)	43.0 (37.8, 48.0)
Men, % (95% CI)	53.7 (50.5, 56.9)	57.9 (51.0, 64.7)	53.4 (51.1, 55.7)	56.0 (51.0, 61.1)
Non-binary, % (95% CI)	1.1 (0.4, 1.9)	0.8 (0.1, 1.4)	0.8 (0.4, 1.2)	0.9 (0.3, 1.5)
AB[1], % (95% CI)	35.9 (32.4, 39.4)	42.2 (36.4, 47.9)	38.2 (36.1, 40.3)	41.7 (39.6, 43.9)
C1C2[1], % (95% CI)	50.8 (47.2, 54.5)	44.9 (39.9, 50.0)	48.2 (45.2, 51.3)	46.4 (43.3, 49.5)
DE[1], % (95% CI)	13.3 (10.9, 15.7)	12.9 (10.0, 15.8)	13.6 (10.3, 16.8)	11.9 (8.9, 15.0)
England, % (95% CI)	87.1 (85.1, 89.1)	88.0 (87.0, 89.0)	85.8 (81.0, 90.6)	84.4 (78.5, 90.3)
Scotland, % (95% CI)	8.6 (7.5, 9.8)	8.5 (7.5, 9.6)	9.5 (6.3, 12.8)	9.9 (6.2, 13.6)
Wales, % (95% CI)	4.3 (3.2, 5.3)	3.5 (2.4, 4.5)	4.7 (2.8, 6.5)	5.7 (3.4, 8.0)
AUDIT-C 5-7, % (95% CI)	54.8 (52.3, 57.3)	63.2 (59.4, 66.9)	57.5 (51.9, 63.1)	67.0 (64.1, 69.8)
AUDIT-C 8-10, % (95% CI)	38.9 (37.6, 40.2)	33.8 (29.9, 37.6)	35.8 (29.9, 41.7)	27.8 (23.9, 31.7)
AUDIT-C 11-12, % (95% CI)	6.4 (4.2, 8.5)	3.0 (1.7, 4.4)	6.7 (4.5, 8.9)	5.3 (3.6, 6.9)

[1]These categories refer to social grades as a measure of socioeconomic position, with AB most advantaged and DE least advantaged. Abbreviations: CI, confidence interval. IQR, interquartile range. Alcohol consumption levels categorised as increasing risk: AUDIT-C 5-7; higher risk: AUDIT-C 8-10; possible dependence: AUDIT-C 11-12.

5. Unweighted characteristics of participants

Table S3: Characteristics of included adult drinking at increasing and higher risk levels making a past-year cut-down attempt ($N_{unweighted}$=9,397, data unweighted).

**Characteristic**	**Unweighted estimate**
Age, median in years (IQR)	50 (36, 61)
Women, % (95% CI)	40.1 (39.1, 41.1)
Men, % (95% CI)	59.2 (58.2, 60.2)
Non-binary, % (95% CI)	0.7 (0.6, 0.9)
Social grades AB, % (95% CI)	31.6 (30.7, 32.6)
Social grades C1C2, % (95% CI)	56.8 (55.8, 57.8)
Social grades DE, % (95% CI)	11.5 (10.9, 12.2)
England, % (95% CI)	70.8 (69.9, 71.7)
Scotland, % (95% CI)	19.1 (18.3, 19.9)
Wales, % (95% CI)	10.1 (9.5, 10.7)
AUDIT-C 5-7, % (95% CI)	59.1 (58.1, 60.0)
AUDIT-C 8-10, % (95% CI)	34.9 (34.0, 35.9)
AUDIT-C 11-12, % (95% CI)	6.0 (5.5, 6.5)
Serious attempt, % (95% CI)	33.1 (32.2, 34.1)

Abbreviations: CI, confidence interval; IQR, interquartile range. Social grades AB indicate most advantaged and DE least advantaged socioeconomic positions. Alcohol consumption levels categorised as increasing risk: AUDIT-C 5-7; higher risk: AUDIT-C 8-10; possible dependence: AUDIT-C 11-12.

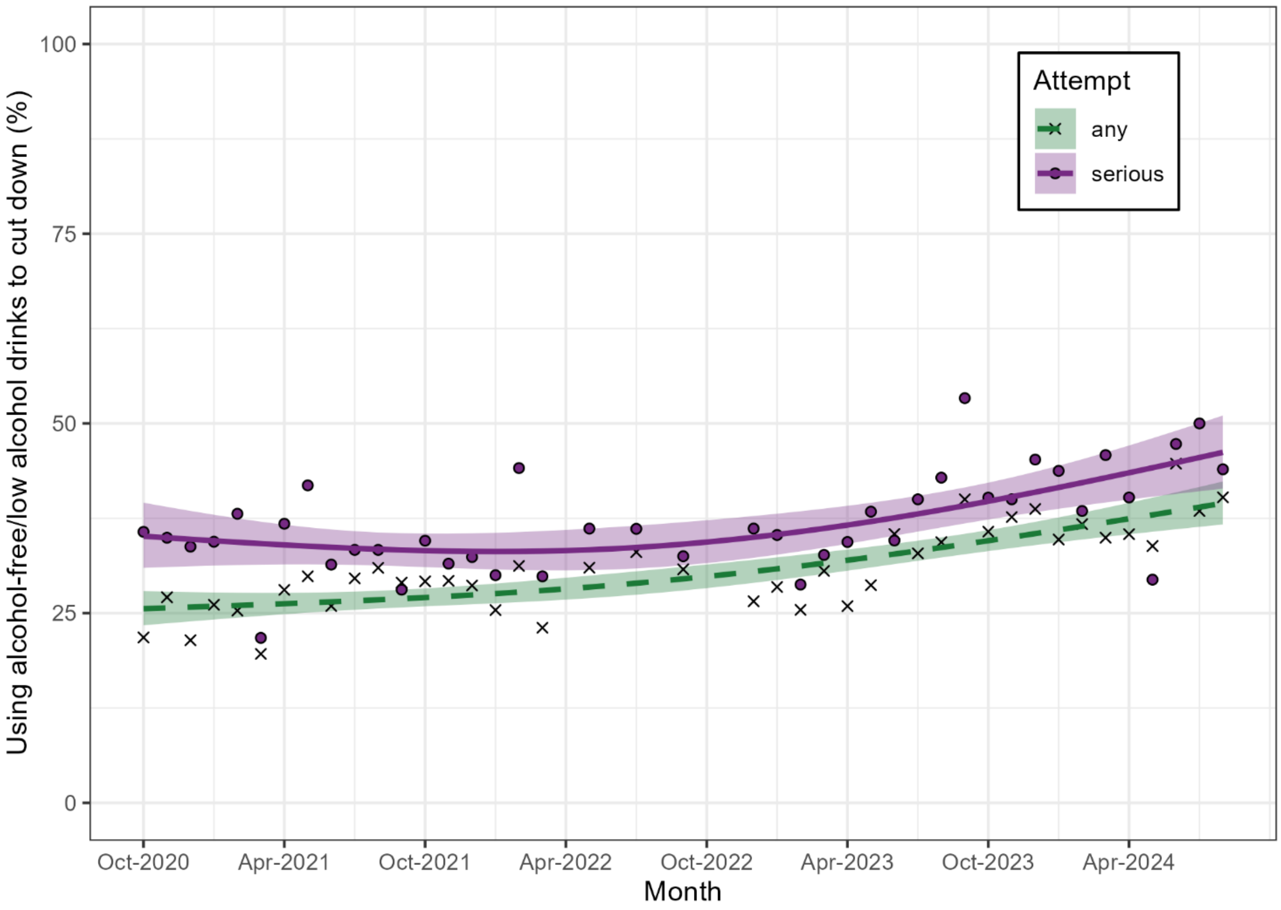

Figure S2: Time trends (2020-2024) in the unweighted prevalence of using alcohol-free/low alcohol drinks in any or serious attempts to reduce alcohol consumption among people drinking at increasing and higher-risk levels who tried to restrict their consumption in the past year, modelled using restricted cubic splines. Shaded areas represent 95% CIs. Dots and crosses represent unmodelled estimates.

Table S4: Unweighted modelled estimates for October 2020 and August 2024 and corresponding prevalence ratios for using alcohol-free/low alcohol drinks to cut down consumption among people drinking at increasing and higher-risk levels in serious or any attempt and in any attempt stratified by sociodemographic and drinking characteristics

	**Oct 20, % (95% CI)**	**Aug 24, % (95% CI)**	**PR (95% CI)**
In serious attempt	35.1 (31.0, 39.6)	46.2 (41.4, 51.0)	1.25 (1.14, 1.38)
In any attempt	25.6 (23.6, 27.9)	39.5 (36.7, 42.3)	1.52 (1.38, 1.67)
Age 18	30.3 (23.5, 38.1)	33.9 (26.4, 42.4)	1.12 (0.84, 1.49)
Age 25	29.7 (25.0, 34.9)	36.6 (31.0, 42.5)	1.23 (0.97, 1.57)
Age 35	28.6 (25.6, 31.8)	40.0 (36.2, 43.9)	1.40 (1.12, 1.74)
Age 45	27.0 (23.9, 30.3)	42.2 (38.2, 46.4)	1.56 (1.26, 1.94)
Age 55	24.6 (21.8, 27.7)	42.3 (38.3, 46.4)	1.72 (1.40, 2.11)
Age 65	21.5 (18.5, 24.9)	40.2 (36.3, 44.2)	1.87 (1.55, 2.25)
Age 75	18.2 (13.4, 24.2)	36.5 (29.9, 43.6)	2.00 (1.58, 2.54)
Women	27.2 (23.8, 30.9)	43.5 (38.9, 48.3)	1.60 (1.37, 1.87)
Men	24.3 (21.5, 27.4)	37.3 (33.8, 40.9)	1.53 (1.35, 1.75)
Social grades AB	28.7 (24.6, 33.1)	44.8 (40.0, 49.6)	1.56 (1.33, 1.83)
Social grades C1C2	24.5 (21.8, 27.6)	37.7 (34.0, 41.6)	1.54 (1.29, 1.83)
Social grades DE	23.2 (17.5, 30.0)	29.7 (22.4, 38.2)	1.28 (1.95, 1.73)
England	25.7 (23.2, 28.4)	40.2 (36.7, 43.8)	1.56 (1.39. 1.75)
Scotland	25.1 (20.2, 30.8)	37.5 (31.8, 43.5)	1.49 (1.25, 1.78)
Wales	25.4 (18.8, 33.4)	40.3 (32.6, 48.5)	1.59 (1.27, 1.98)
AUDIT-C 5-7	26.0 (23.1, 29.1)	42.4 (38.7, 46.1)	1.63 (1.44, 1.84)
AUDIT-C 8-10	25.6 (22.1, 29.4)	34.7 (30.1, 39.5)	1.35 (1.14, 1.61)
AUDIT-C 11-12	21.6 (14.3, 31.2)	37.5 (26.8, 49.7)	1.74 (1.26, 2.40)

Abbreviations: CI, confidence interval. Social grades AB indicate most advantaged and DE least advantaged socioeconomic positions. Alcohol consumption levels categorised as increasing risk: AUDIT-C 5-7; higher risk: AUDIT-C 8-10; possible dependence: AUDIT-C 11-12.

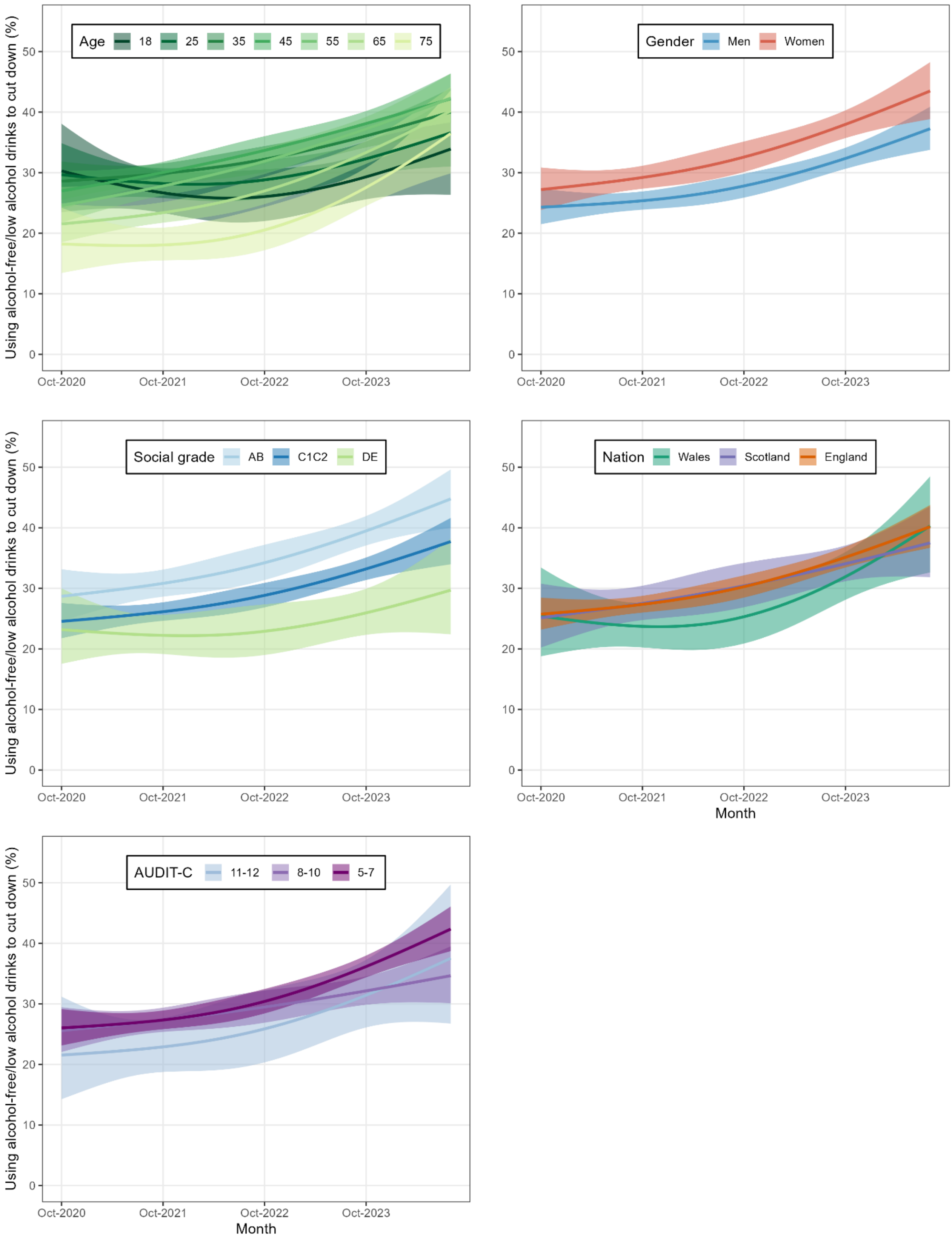

Figure S3: Time trends (2020-2024) in the prevalence of using alcohol-free/low alcohol drinks to reduce alcohol consumption among people drinking at increasing and higher-risk levels who tried to restrict their consumption in the past year, stratified by age, gender, social grade, nation, and alcohol consumption level. Social grades AB indicate most advantaged and DE least advantaged socioeconomic positions. Alcohol consumption levels categorised as increasing risk: AUDIT-C 5-7; higher risk: AUDIT-C 8-10; possible dependence: AUDIT-C 11-12. Shaded areas represent 95% CIs.

Table S5: Sociodemographic and drinking profile of people drinking at increasing and higher-risk levels who used alcohol-free/low alcohol drinks to reduce alcohol consumption across the years.

	**Oct 20 – Sep 21**	**Oct 21 – Sep 22**	**Oct 22 – Sep 23**	**Oct 23 – Aug 24**
Age, median (IQR)	47 (34, 57)	46 (35, 57)	51 (39, 61)	50 (37, 63)
Women, % (95% CI)	46.7 (44.8, 48.5)	40.9 (39.1, 42.7)	44.7 (42.9, 46.6)	40.7 (38.9, 45.2)
Men, % (95% CI)	52.2 (50.4, 54.0)	58.3 (56.5, 60.1)	54.5 (52.6, 56.3)	58.4 (56.6, 60.2)
Non-binary, % (95% CI)	1.1 (0.7, 1.5)	0.8 (0.5, 1.1)	0.8 (0.5, 1.1)	0.9 (0.5, 1.2)
AB[1], % (95% CI)	31.4 (29.7, 33.2)	36.5 (34.8, 38.3)	36.5 (34.7, 38.2)	41.2 (39.4, 43.0)
C1C2[1], % (95% CI)	57.7 (55.9, 59.5)	54.9 (53.0, 56.7)	54.9 (53.1, 56.8)	50.5 (48.7, 52.3)
DE[1], % (95% CI)	10.8 (9.7, 12.0)	8.6 (7.6, 9.6)	8.6 (7.6, 9.6)	8.3 (7.3, 9.3)
England, % (95% CI)	74.2 (72.6, 75.8)	75.5 (73.9, 77.1)	68.6 (66.9, 70.3)	66.8 (65.1, 68.5)
Scotland, % (95% CI)	17.0 (15.6, 18.4)	17.5 (16.1, 18.9)	21.3 (19.8, 22.8)	21.4 (19.9, 22.9)
Wales, % (95% CI)	8.8 (7.8, 9.8)	7.0 (6.0, 7.9)	10.0 (8.9, 11.1)	11.9 (10.7, 13.0)
AUDIT-C 5-7, % (95% CI)	56.4 (54.5, 58.2)	63.3 (61.5, 65.1)	58.9 (57.1, 60.7)	65.7 (63.9, 67.4)
AUDIT-C 8-10, % (95% CI)	37.6 (35.8, 39.4)	33.1 (31.4, 34.8)	34.9 (33.1, 36.6)	29.3 (27.6, 30.9)
AUDIT-C 11-12, % (95% CI)	6.0 (5.2, 6.9)	3.6 (2.9, 4.3)	6.2 (5.3, 7.1)	5.1 (4.3, 5.9)

[1]These categories refer to social grades as a measure of socioeconomic position, with AB most advantaged and DE least advantaged.

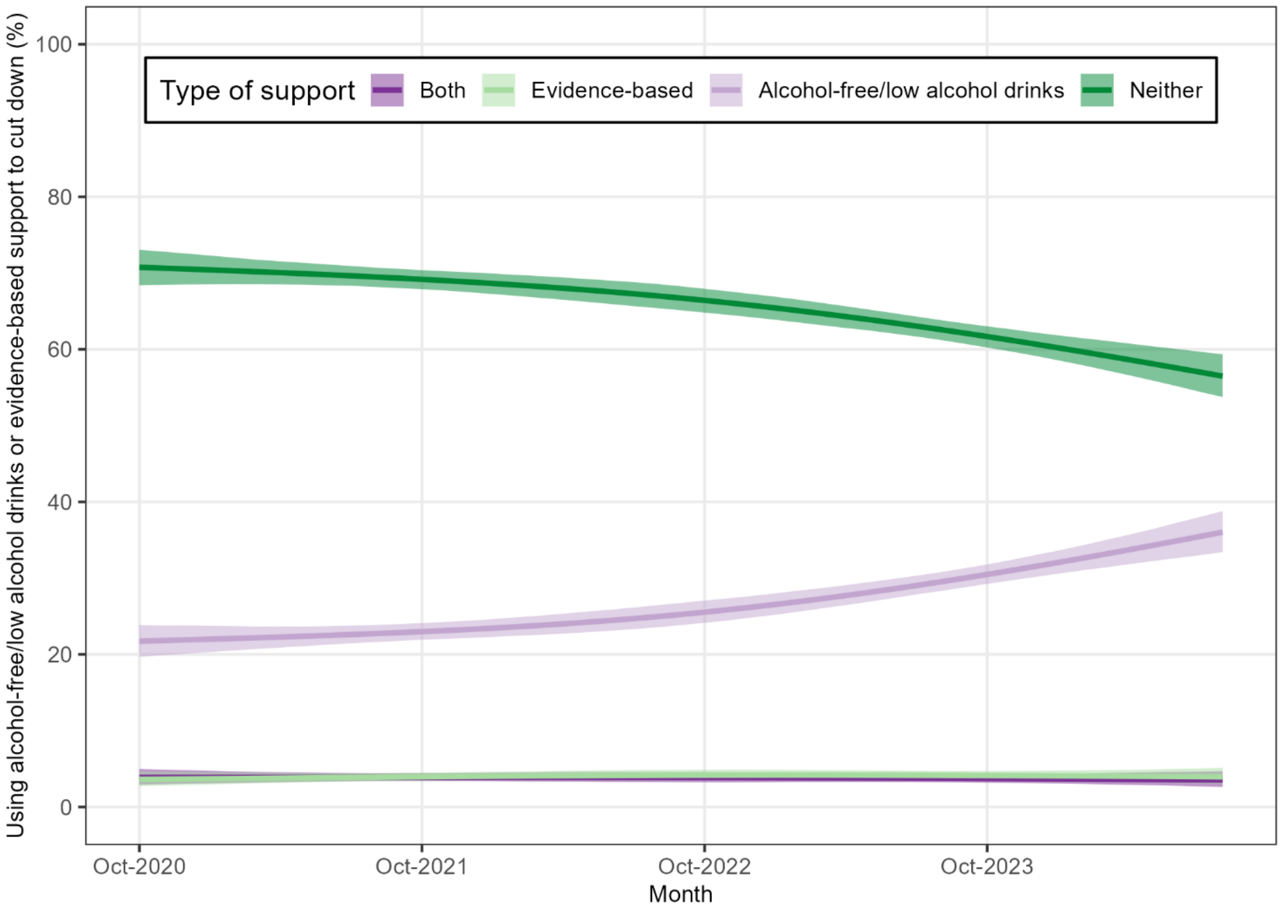

Figure S4: Time trends (2020-2024) in the proportion of people drinking at increasing and higher-risk levels who tried to restrict their consumption in the past year by using (i) alcohol-free/low alcohol drinks and evidence-based support; (ii) alcohol-free/low alcohol drinks but not evidence-based support; (iii) evidence-based support but not alcohol-free/low alcohol drinks; or (iv) neither. Shaded areas represent 95% CIs.
